# An Energy Model of Place Cell Network in Three Dimensional Space

**DOI:** 10.3389/fnins.2018.00264

**Published:** 2018-04-25

**Authors:** Yihong Wang, Xuying Xu, Rubin Wang

**Affiliations:** Science School, East China University of Science and Technology, Shanghai, China

**Keywords:** place cells, three dimensional space, energy coding, spatial selectivity, locating system

## Abstract

Place cells are important elements in the spatial representation system of the brain. A considerable amount of experimental data and classical models are achieved in this area. However, an important question has not been addressed, which is how the three dimensional space is represented by the place cells. This question is preliminarily surveyed by energy coding method in this research. Energy coding method argues that neural information can be expressed by neural energy and it is convenient to model and compute for neural systems due to the global and linearly addable properties of neural energy. Nevertheless, the models of functional neural networks based on energy coding method have not been established. In this work, we construct a place cell network model to represent three dimensional space on an energy level. Then we define the place field and place field center and test the locating performance in three dimensional space. The results imply that the model successfully simulates the basic properties of place cells. The individual place cell obtains unique spatial selectivity. The place fields in three dimensional space vary in size and energy consumption. Furthermore, the locating error is limited to a certain level and the simulated place field agrees to the experimental results. In conclusion, this is an effective model to represent three dimensional space by energy method. The research verifies the energy efficiency principle of the brain during the neural coding for three dimensional spatial information. It is the first step to complete the three dimensional spatial representing system of the brain, and helps us further understand how the energy efficiency principle directs the locating, navigating, and path planning function of the brain.

## Introduction

The spatial cognition function is one of the most important functions of the brain. Many types of cells are contributing to the locating and navigating function. Among them, place cells in hippocampus and grid cells in entorhinal cortex are the most fundamental and well-studied cells. The colorful researches during the last few decades have revealed the representational function of place cells in the hippocampus. Spatial receptive fields of spiking neurons in the rat hippocampus are firstly reported by O'Keefe and Dostrovsky (1971). When the rat was in a certain place in the local environment, these place cells fired intensively. The set consists of these responding spatial locations is named the place field of a cell. Different cells firing at different locations, as a result, the environment is represented by place cells population in hippocampus (Wilson and McNaughton, 1993). And a same place cell can participate in representations for different environments. Representation of environment by place cells can be updated in a dynamic, continuous manner. Furthermore, place cell is the component of a more general circuit dynamically representing the spatial information (Moser et al., 2008). Resent experimental and modeling works find out that in large environments, place cells in hippocampus express multiple place fields (Park et al., 2011; Pilly and Grossberg, 2013; Hedrick and Zhang, 2016). Another important part of this system is the grid cell. It is found in the medial entorhinal cortex (MEC) and neighboring limbic structures. Grid cells have firing fields like the place fields, but the fields are multiply periodical which regularly show a triangular grid pattern (Fyhn et al., 2004; Hafting et al., 2005; Sargolini et al., 2006; Hasselmo et al., 2007). MEC grid cells project to hippocampal place cells and are hypothesized to play a role in path integration (Hafting et al., 2005; Barry et al., 2007). The firing rate changes of grid cell may affect the place field of the place cell (Kubie and Fox, 2015). The coordinate type of the grid indicates grid cells as possible elements of a metric system for spatial navigation (Hafting et al., 2005). Place cells and grid cells form a quantitative spatial-temporal representation system for location, path, and associated behavior, experience and memory. Because these cells have remarkable activity patterns in non-sensor systems, their firing structures may reflect the internal operations of the system. The study of place cells, grid cells and the spatial representation system of the brain can provide a deeper understanding of cortical network dynamics (Yates, 2013; Hayakawa et al., 2015; Pfeiffer and Foster, 2015; Bechtel, 2016; Geiller et al., 2017; Kentros et al., 2017; Scaplen et al., 2017; Trimper et al., 2017).

Since the discovery of place cells, many models have attempted to explain how this spatial selectivity arises within the hippocampus (Samsonovich and McNaughton, 1997; Hartley et al., 2000; Káli and Dayan, 2000) and how the place fields are formed. Their influence on navigation remains an important experimental and theoretical question. Particularly, little is known on how different sensory cues contribute to place field formation and spatial navigation (Kulvicius et al., 2008). During the four decades' researches, different models have been proposed for hippocampal place cell formation including Gaussian functions (O'Keefe and Burgess, 1996; Touretzky and Redish, 1996; Foster et al., 2000; Hartley et al., 2000), back-propagation algorithm (Shapiro and Hetherington, 1993), auto-associative memory (Recce and Harris, 1996), competitive learning (Sharp, 1991; Brown and Sharp, 1995), neural architecture based on landmark recognition (Gaussier et al., 2002), neuronal plasticity (Arleo and Gerstner, 2000; Arleo et al., 2004; Krichmar et al., 2005; Sheynikhovich et al., 2005; Strösslin et al., 2005), independent component analysis (Takács and Lorincz, 2006; Franzius et al., 2007), self-organizing map (Chokshi et al., 2003; Ollington and Vamplew, 2004), Kalman filter (Bousquet et al., 1998; Balakrishnan et al., 1999), and odor supported model(Kulvicius et al., 2008). However, neither these theoretical researches nor other experimental researches have focused on a basic but important question, which is how the real three dimensional space is represented by place cells. Almost all the animals are living in the three dimensional world while the research of place cells remain in one (a line) or two (a plane) dimensional space. Only a few known studies reported how the three dimensional space is recognized by animals (Hayman et al., 2011; Rowland and Moser, 2015). Unfortunately, these results seemed to be contradictory. Hayman believed that the animal applied different strategies in coding the horizontal and vertical spatial information (Hayman et al., 2011), suggesting an asymmetric coding property about three dimensional space. Whereas, Moser supported that the place code for the three dimensions is symmetric (Rowland and Moser, 2015). Obviously, this question has not been studied thoroughly and the known results indicated that it is hard to reveal the mystery of the three dimensional spatial cognition function only by experimental studies. Theoretical modeling should also be addressed to solve this problem. The difficulties of conducting an experiment in three dimensional space are the limitation of experimental techniques and data recording methods. They may be the main reasons why the study is insufficient. For example, it is hard to record data by electrodes in the brain when a bat or bird is flying, a monkey is climbing or a fish is swimming. Due to these limitations in studying place cells mentioned above, it is important and convenient to investigate this question theoretically in advance, and if the model proposed is reasonable, it can provide guidance and predictions for future experimental and theoretical studies.

The spatial representation of place cells is essentially a neural information coding problem, which has been the core problem in cognitive neural science (Amari and Hiroyuki, 2005; Gazzaniga et al., 2009). The electrical activity of neuron is the basis of neural coding. Classic coding theories such as phase coding, frequency coding and group coding describe the electrical activity of neurons by action potential or firing rate. Unfortunately, these techniques are limited in scope and are difficult to accomplish the global coding successfully (Borst and Theunissen, 1999; Purushothaman and Bradley, 2005; McLaughlin, 2009). Currently, no complete theory for neural coding and decoding can direct the research of global brain activities. The main reasons are that the cross-level influence of large-scale neural activities are too complicated and the neurodynamics are nonlinear. These properties will make it hard to perfectly analyze the neural coding and decoding problem (Laughlin and Sejnowski, 2003; Singer, 2009). The major goal of neural code is to represent information. However, under the selective pressure, the neural system of the animal must make the best use of the energy. Both experimental and computational evidences suggest that neural systems may maximize the efficiency of energy consumption in processing neural signals and neural code should be energy efficient (Yu and Yu, 2017). So it is reasonable to regard the energy efficiency as a constraint to the neural systems. A new alternative called energy coding theory argues that neural information can be expressed by neural energy, so that the neural information processing can be placed within the framework of the global neural coding of the brain (Wang R. et al., 2015; Wang Z. et al., 2015). Neural networks are difficult to model and analyze because they are high-dimensional nonlinear dynamical systems which composed of large number of neurons. Nevertheless, the superimposing property of neural energy can provide considerable convenience for neural modeling and computational analyzing, which will greatly reduce the cost of analytical research (Wang R. et al., 2015). Furthermore, energy is a more fundamental variable than others such as spike number, firing rate or oscillation phase. So neural energy may be an effective tool to study the global activity of the brain. However, the research of neural energy coding is still in its infancy. Although the energy properties of a single neuron and the structural network have been surveyed (Wang R. et al., 2015; Wang Z. et al., 2015), the appropriate functional network model has not been fully established and studied. So the energy method has not been used in modeling a certain cognitive function of neural systems. Meanwhile, due to current technical constraints, the corresponding experimental data for neural energy are scarce.

Due to the defects mentioned above, it is quite necessary to study the three dimensional spatial representation function of place cells by the energy method. In this research, we constructed a network model for place cells to represent three dimensional space on an energy level. The cells which have various place fields achieved accurate locating function. Then we analyzed the energy consumption properties and locating errors under the situations of different field sizes. The results have shown that this model captured the basic behaviors of place cells and revealed the energy efficiency property of the neural system.

## Model and methods

### The energy consumption of place cells

It is very difficult to directly measure the energy consumption of a cell due to the limitation of current recording techniques. However, it is possible to calculate the energy consumption of a cell based on a proper model describing the ion currents (Laughlin et al., 1998; Attwell and Laughlin, 2001; Crotty et al., 2006; Moujahid et al., 2011). Notably, energy is supplied to ion pump by the metabolism of adenosine triphosphate (ATP). The energy is primarily used to transport the ions against the ion concentration gradient. During electrical activity of a neuron, ions are driven by concentration gradient to cross the cell membrane and form the ion currents. The Joule heat due to resistances of ion channel conductance can be a convenient approach to understand neural energy based on an equivalent electrical circuit of neurons. The Hodgkin-Huxley (H-H) Model is the most successful model on the ion channel level. So the neural energy can be calculated by H-H model.

The equations of H-H model are:

$$\begin{array}{l} C_{m}\frac{dV_{m}}{dt}=g_{l}(E_{l}-V_{m})+g_{Na}m^{3}h(E_{Na}-V_{m})\\ \;+g_{K}n^{4}(E_{K}-V_{m})+I \end{array}$$

$$\{\begin{array}{l} \frac{dn}{dt}=\alpha_{n}(1-n)-\beta_{n}n\\ \frac{dm}{dt}=\alpha_{m}(1-m)-\beta_{m}m\\ \frac{dh}{dt}=\alpha_{h}(1-h)-\beta_{h}h \end{array}$$

(1) $$\begin{array}{l} \alpha_{n}=\frac{0.01(10+V_{m}-V_{r})}{\text{exp}\left[((10+V_{m}-V_{r})/10)-1\right]}\;\beta_{n}=0.125\text{exp}(\frac{V_{m}-V_{r}}{80})\\ \alpha_{m}=\frac{0.1(25+V_{m}-V_{r})}{\text{exp}\left[((25+V_{m}-V_{r})/10)-1\right]}\;\beta_{m}=4\text{exp}(\frac{V_{m}-V_{r}}{18})\\ \alpha_{h}=0.07\text{exp}(\frac{V_{m}-V_{r}}{20})\;\beta_{h}=\frac{1}{\text{exp}\left[(30+V_{m}-V_{r})/10+1\right]} \end{array}$$

where *C*_*m*_ is membrane capacitance of a neuron, *V*_*m*_ is membrane potential, *E*_*Na*_ and *E*_*K*_ are Nernst potentials of Na^+^ and K^+^, and *E*_*l*_ is the potential while there is no leakage current. *g*_*l*_*, g*_*Na*_, and *g*_*K*_ are, respectively, the leakage conductance, Na^+^ channel conductance, and K^+^ channel conductance. The typical values of these parameters are: resting membrane potential *V*_*r*_ = 67.3 mV, maximum Na^+^ conductance *g*_*Na*_ = 120 mS/cm^2^, maximum K^+^ conductance *g*_*K*_ = 36 mS/cm^2^, leakage conductance *g*_*l*_ = 0.3 mS/cm^2^, and Nernst potentials are 50, −80, and −56 mV, respectively. Based on H-H model, we can theoretically calculate the energy consumption of neuronal activity. The energy consumed by a neuron during a certain period of time can be deduced. The equation is shown as follows (Laughlin et al., 1998; Attwell and Laughlin, 2001; Crotty et al., 2006; Moujahid et al., 2011; Wang et al., 2017a),

(2) $$\begin{array}{l} E_{c}=\int_{t}[V_{m}I+i_{Na}(E_{Na}-V_{m})+i_{K}(E_{K}-V_{m})\\ \;+i_{l}(E_{l}-V_{m})]dt \end{array}$$

Then it can be calculated that ~1.88 × 10^−7^ J energy is consumed by a typical neuron during an action potential (Wang et al., 2017a). This value, which can transfer the number of spikes into energy consumption, will be embedded into the network model later to determine the power and energy consumed by the place cell network. Then we can analyze the energy properties of the place cell network during three dimensional space exploration and localization.

### Experiment environment and neural network model

**Figure 1:**
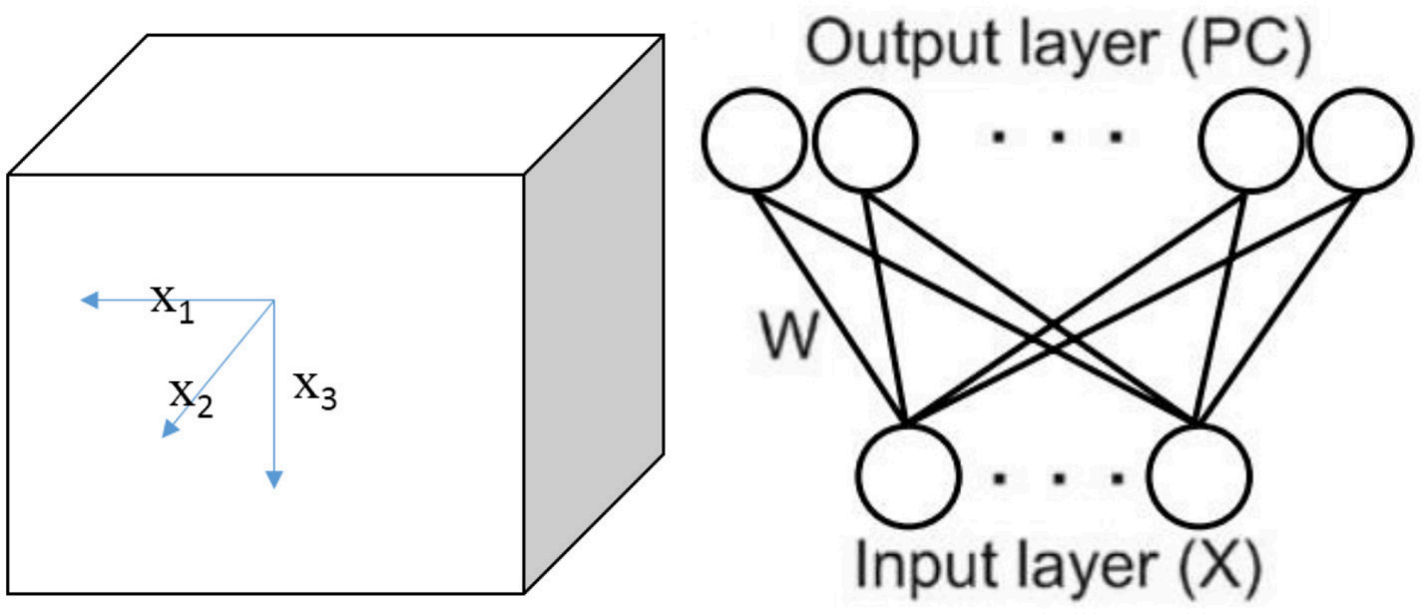
Simplified three dimensional space model and the neural network.

We set up a cube space with a side length of L (see Figure 1). A bat or bird is placed randomly in a position of the environment, setting a task of learning the environment in the experiment. In this cube space, six borders are regarded as landmarks. The animal perceives environmental cues by using its visual (bird) or auditory (bat) system, then learns the sensory information by its neural network made of place cells.

### Sensory model

In the experiment, the environment is a cube place without any references other than the borders, where the animal can only get visual or auditory information from six walls (landmarks). We refer to the six walls as front (F), back (B), left (L), right (R), up (U), and down (D). When study the locating function of place cells in two dimensional space, researchers (O'Keefe and Burgess, 1996; Ollington and Vamplew, 2004) chose the distances to the four walls (East, West, North, and South) as the input to the sensory. We use the similar method but generalize it in the three dimensional space. Meanwhile, since only three variables among the six distances from the borders are independent, we choose the distances from L, F, and D walls as the independent inputs to the sensory.

Note that the actual location of the animal at time t is described by vector X(t) = (x_1_(t), x_2_(t), x_3_(t)), where x_i_(t) is the actual distance from wall *i*(*i* = 1, 2, 3) corresponding to L, F, D (Figure 1). However, it is important and reasonable to assume that sensory neurons of the animal are unable to acquire accurate perception of its own locations. So the perception input model is given by the following equation:

(3) $$\{\begin{array}{l} X{\left(t\right)}^{\prime }=\left(x_{1}{\left(t\right)}^{\prime },x_{2}{\left(t\right)}^{\prime },x_{3}{\left(t\right)}^{\prime }\right)\\ x_{i}{\left(t\right)}^{\prime }=x_{i}\left(t\right)\left(1+\alpha \eta \right),i=1,2,3 \end{array}$$

where α represents the error rate of the sensory (visual or auditory) perception, which is dependent on the individual animal. η is a random number from a uniform distribution within the interval [−1, 1], that is η~U(−1,1) (Yan et al., 2016).

### Energy model for place cells and learning rule

The place cells are considered to receive the geometric inputs of boundary vector cells, each of which responds when a boundary is at a particular distance to the animal (Hartley et al., 2000; Kulvicius et al., 2008). So the firing of place cells can be seen as the sum of inputs received from boundary vector cells (Kulvicius et al., 2008). And these boundary vector cells perform the sensory function. Then a feed-forward network can be applied to construct a model to describe the locating system constituted of place cells and sensor neurons, similar as the studies in two dimensional space (O'Keefe and Burgess, 1996; Hartley et al., 2000; Kulvicius et al., 2008; Yan et al., 2016). Sensory neurons input to place cells with X (t)′. This is a fully-connected network between layers where every sensory neuron is connected to every place cell with weights matrix W (t) = [w_*ij*_(t)]_3×N_, where *i* = 1, 2, 3 represent three sensory neurons, j = 1, 2, …, N represent the N place cells. Three sensory neurons perceive the distances from boundary L, F, and D. W (t) is a 3 × N matrix, and w_ij_(t) is the connection weight from the ith sensory neuron to the jth place cell at time t. Weights are initialized by the following functions (Kulvicius et al., 2008),

(4) $$w_{ij}(0)={\left(1+exp(\frac{\;\gamma -E(\gamma )}{2\sigma^{2}})\right)}^{-1}$$

**Figure 2:**
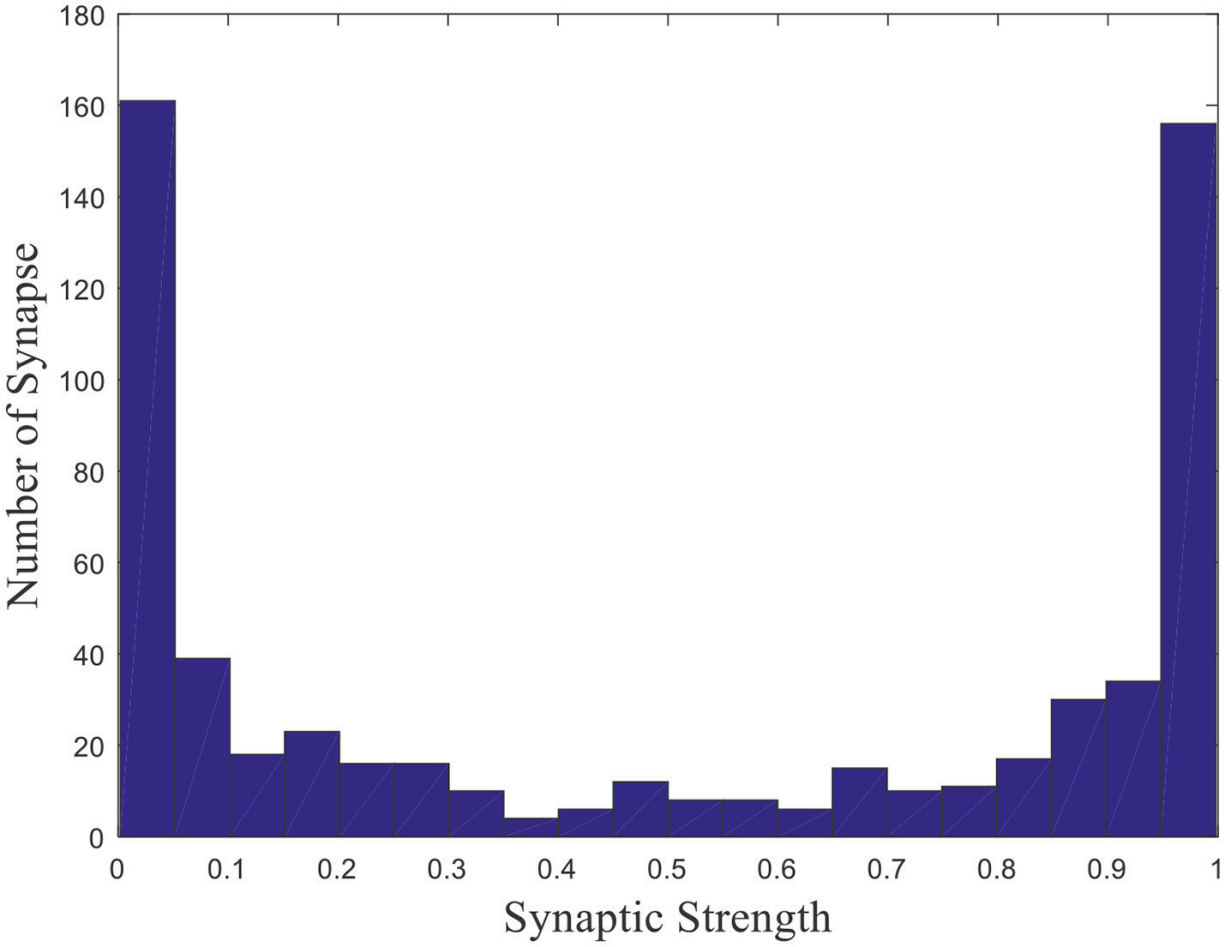
Initial weights distribution.

Where γ is a random number uniformly distributed on the interval [0, 1], that is γ ~ U (0, 1). *E*(γ) which equals to 0.5 is the expectation of the uniformly distributed random number γ. The weights distribution is shown in Figure 2 after initialization (Kulvicius et al., 2008).

This is a histogram of the initial weights distribution. The horizontal axis represents the synaptic strength, and the vertical axis represents the number of synapse with the corresponding strength. Since γ is uniformly distributed and Equation (4) is central symmetric, the distribution of synaptic strength is symmetric about 0.5. Such a distribution rather than a uniform distribution is that all place field centers will be located around the center of the environment and the model will fail to obtain place fields near the boundary of the environment if the uniform distribution is applied (Kulvicius et al., 2008). Weights are the basis vectors in the model, which are used to compute firing powers of place cells. When competitive learning rule is employed, place cells become tuned to a specific input, which leads to the spatial selectivity.

Inspired by the firing rate model in two dimensional space proposed by O'Keefe and Burgess (1996) and Hartley et al. (2000), we construct the following model which combines the neural energy method to represent three dimensional space as follows,

(5) $$P_{j}(t)=CR_{m}\text{exp}(-{\frac{\left(\frac{1}{n}\left\|\frac{X(t{)}^{\prime }}{L}-W_{j}(t)\right\|\right)}{2\sigma_{j}^{2}}}^{2})$$

Where *P*_*j*_*(t)* is the firing power of the *j*th place cell at time *t, C* is the energy consumption by a place cell during an action potential. As introduced earlier, ~188 nJ energy is consumed to transmit a spike. In order to reflect the diversity of place cells' metabolic environment, *C* is normally distributed from N (188, 10) nJ. *R*_*m*_ is the maximum firing rate of a single place cell, which is about 20 Hz (Hartley et al., 2000). n is the number of sensory inputs and *W*_*j*_*(t)* is the *j*th row of *W(t)*. The norm is the Euclidean distance. And σ_j_ is a random number from a normal distribution which initially affects the range of place field. As a result, the random number σ_j_~N (0.03, 0.005) is another parameter to reflect the diversity of place cells.

According to a classical learning rule with a winner-takes-all mechanism, the connections to the cell with the maximal firing rate wins the learning chance (Kulvicius et al., 2008). Obviously, efficiency of this mechanism is low because only the weights of one winning cell are changed at every step. Meanwhile, the firing rate learning model is inconvenient to generalize to multiple levels. Therefore, learning rule is modified not only in a batch manner (Yan et al., 2016) but also on an energy level as follows,

(6) $$\begin{array}{l} \frac{dW_{J}(t)}{dt}=\mu (\frac{X(t{)}^{\prime }}{L}-W_{J}(t))\\ J=\left\{j|P_{j}(t)>P_{thr}\right\} \end{array}$$

Where μ is the learning rate, *P*_*thr*_ is the responding threshold represents the minimum firing power of cells that are activated. *J* is the response set. And every place cell responding to the current location with a firing power above threshold will modify the weights from sensory neurons.

The firing power of place cell *j* can also be viewed as the function of spatial location *X(t)*′ according to Equation (5). Then the place field of cell j can be defined as the set of all the location *X(t)*′ with firing power larger than *P*_*thr*_. After firing powers are calculated, place field centers can be defined by analyzing the positions within the corresponding place fields. Furthermore, the center of place field related to cell *j* is defined during this dynamical process as follows,

(7) $$C_{j}=\frac{\int_{0}^{+\infty }P_{j}(t)X(t{)}^{\prime }dt}{\int_{0}^{+\infty }P_{j}(t)dt}$$

Then the location of the animal will be estimated by the weighted average of place field centers according to the response set:

(8) $$\begin{array}{l} Loc(t)=\frac{\sum_{J}P_{j}(t)C_{j}}{\sum_{J}P_{j}(t)}\\ J=\left\{j|P_{j}(t)>P_{thr}\right\} \end{array}$$

Where, *Loc(t)* is the location of the animal at moment t determined by this locating model. And *C*_*j*_ is the place field center of cell *j*, while *P*_*j*_*(t)* is the activity power of cell *j* at moment *t*.

## Results

### Energy consumption of an action potential of a place cell

According to the described method, we calculate the neural energy consumed by place cell firing an action potential and perform the numerical simulation first.

**Figure 3:**
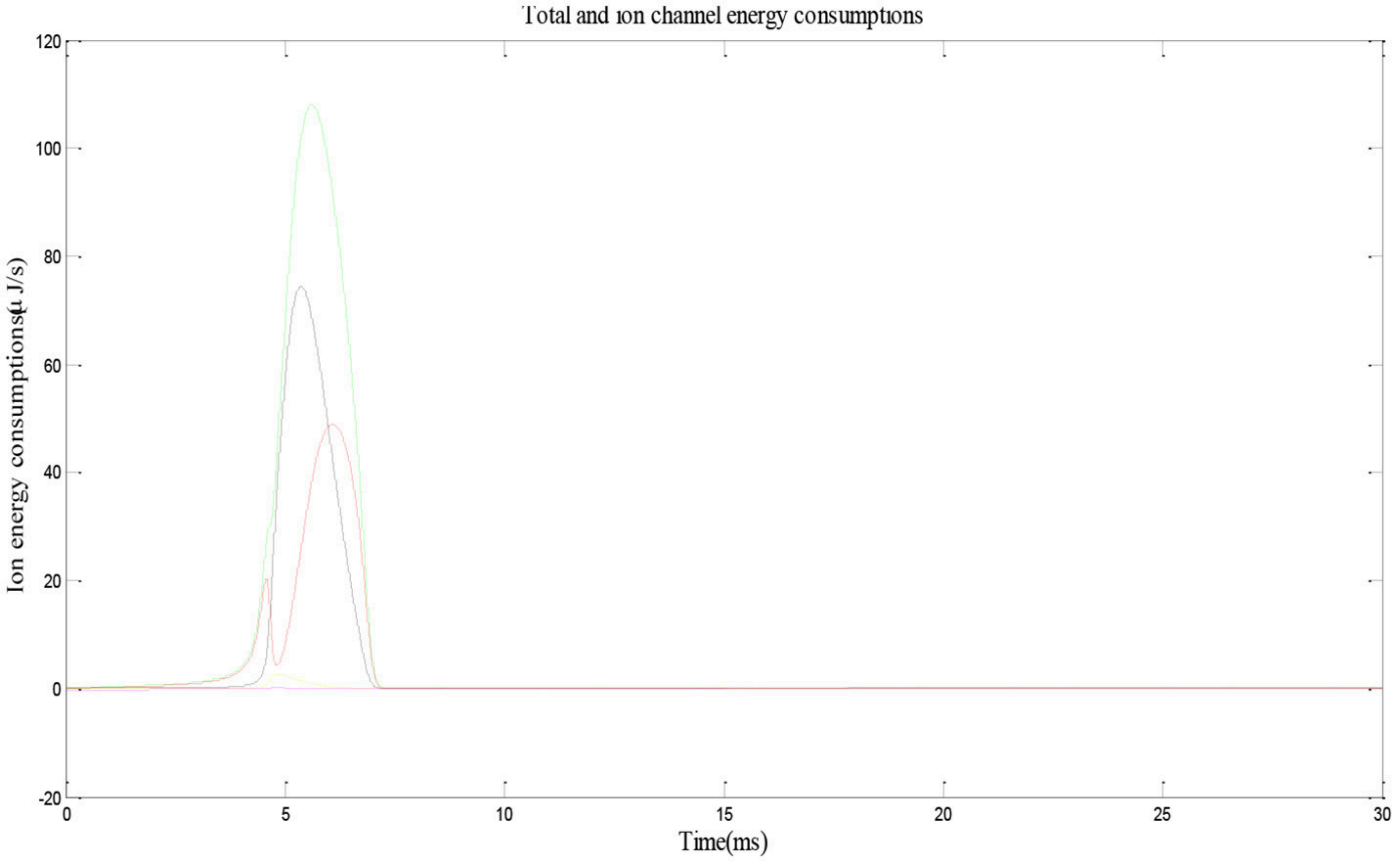
Energy consumption by a place cell during an action potential (Wang et al., 2017a).

Figure 3 shows the electric power of each ion channel during an action potential of a place cell. Green line is the total power and red, black, yellow, and fuchsia lines are powers of Na^+^, K^+^, leakage, and stimulus currents, respectively. By integrating the power over time, we can get that one action potential costs about 188 nJ energy.

### Exploration results in three dimensional space

**Figure 4:**
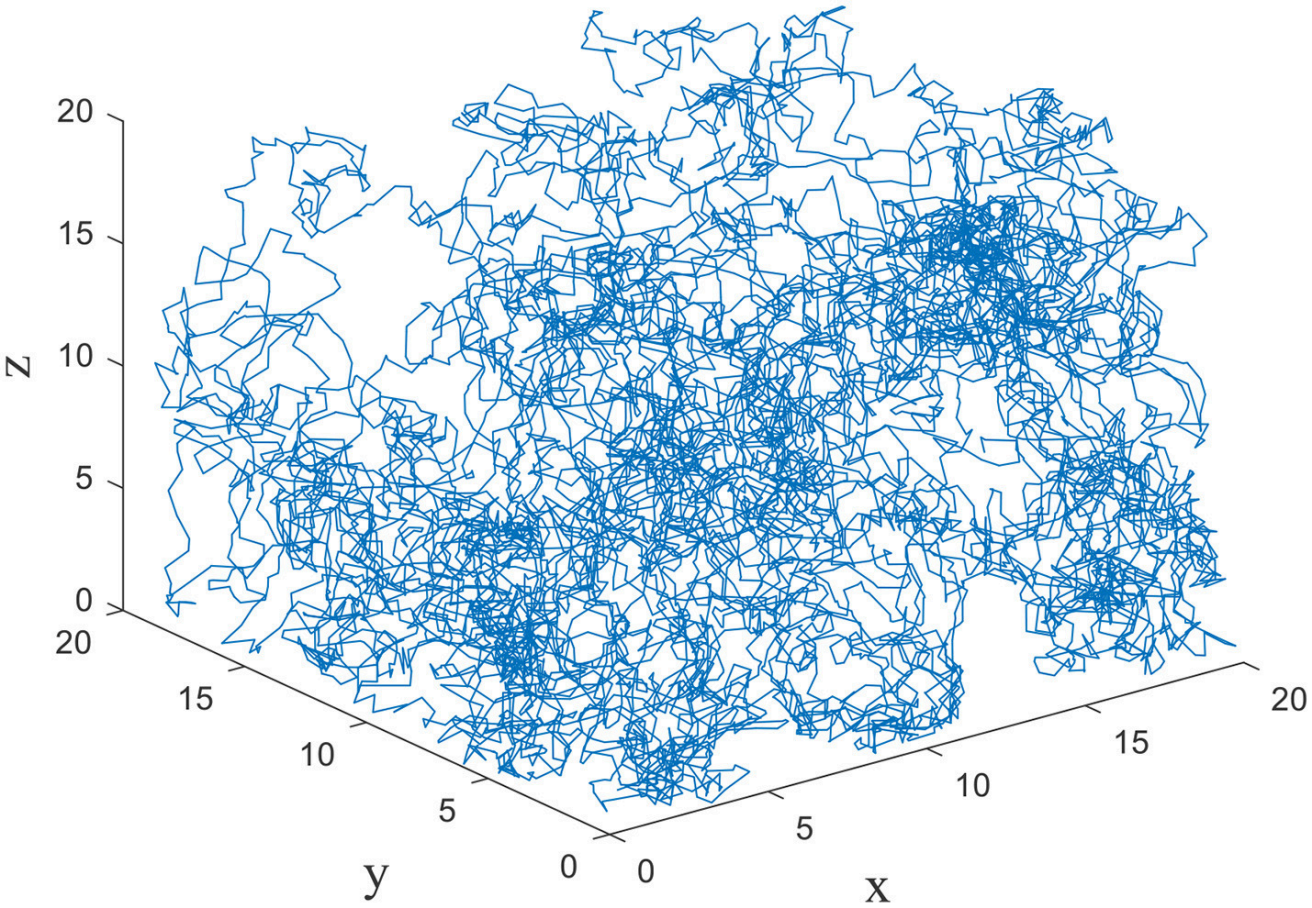
Random exploration trajectory in three dimensional space.

The exploration is performed in three dimensional space by the model. The size of the cube space is 20 × 20 × 20 units, number of place cells is 200, number of sensory neurons is 3, sensory error rate α = 0.1, learning rate μ = 0.001, maximum firing rate R_m_ = 20 Hz, and P_thr_ = 0.3 P_m_, where P_m_ is the maximum firing power. During the experiment, the animal initiated the random search in the cube space. The number of steps is set to be 10,000, and step length is 1. The trajectory of one random search is shown in Figure 4. The coordinates of three dimensions are labeled as x, y, and z. The highly random trajectory covered most of the cube space. This behavior is reasonable for an animal in the absence of food or water reward. Due to the different initial weights, the preliminary responses of the place cells are not the same. Then according to the batch competitive learning rule, each place cell acquires unique spatial selectivity, and form its own place field.

### Various three dimensional spatial tunings of place cells

After the spatial exploration and learning, place cells activities are tuned to specific spatial locations to form the place fields.

**Figure 5:**
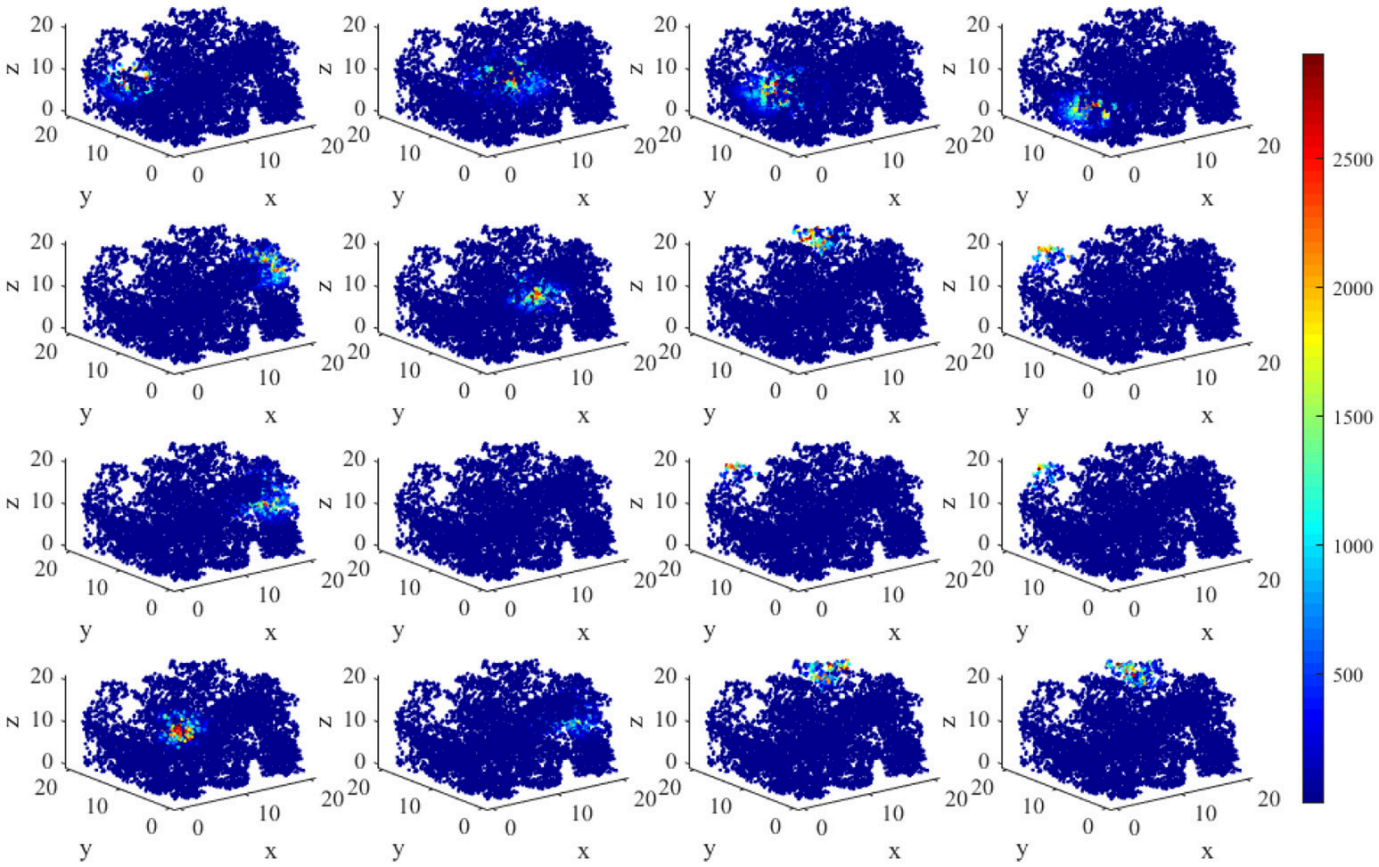
Firing powers of place cells in three dimensional space.

**Figure 6:**
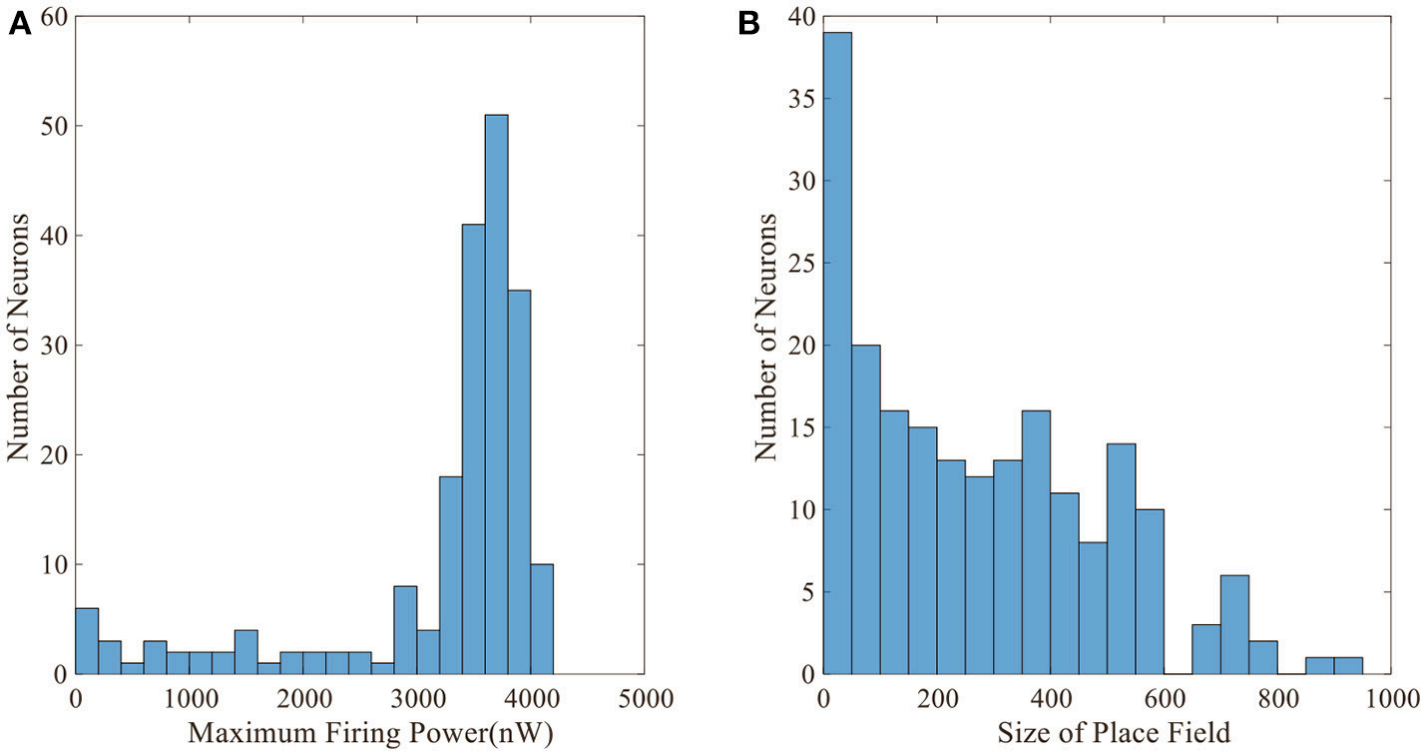
Distributions of maximum firing power and size of place field. **(A)** Maximum firing power distribution among 200 cells. Vertical axis is number of neurons and horizontal axis is maximum firing power. **(B)** Size distribution of place fields among 200 cells. Vertical axis is number of neurons and horizontal axis is size of place field. The size is represented by the number of locations at which the cell is activated.

Figure 5 displays the activity patterns of 16 randomly selected cells. The scatter plots in space represent the different locations of the animal in the random search trajectory, and the colors indicate the firing power of place cells at the corresponding position. We can further know if a certain location belongs to the place field of a cell, that is, whether the cell has spatial selectivity for this location. It can be seen from Figure 5, due to individual differences of place cells, the distribution and size of place fields as well as firing powers vary among different place cells. These facts can further be obtained from the histograms in Figure 6 which illustrates the distributions of maximum firing power (left) as well as the size of place field (right) among the 200 place cells. The vertical axes both represent number of neurons and the horizontal axes represent maximum firing power and size of place field respectively. Note that the size of place field are reflected by the count of locations at which the cellular activity is above threshold. Maximum power is about 3,000 nW among these 16 random selected cells in Figure 5. Larger place fields usually have higher maximal power. This may be the consequence of competitive learning rule.

**Figure 7:**
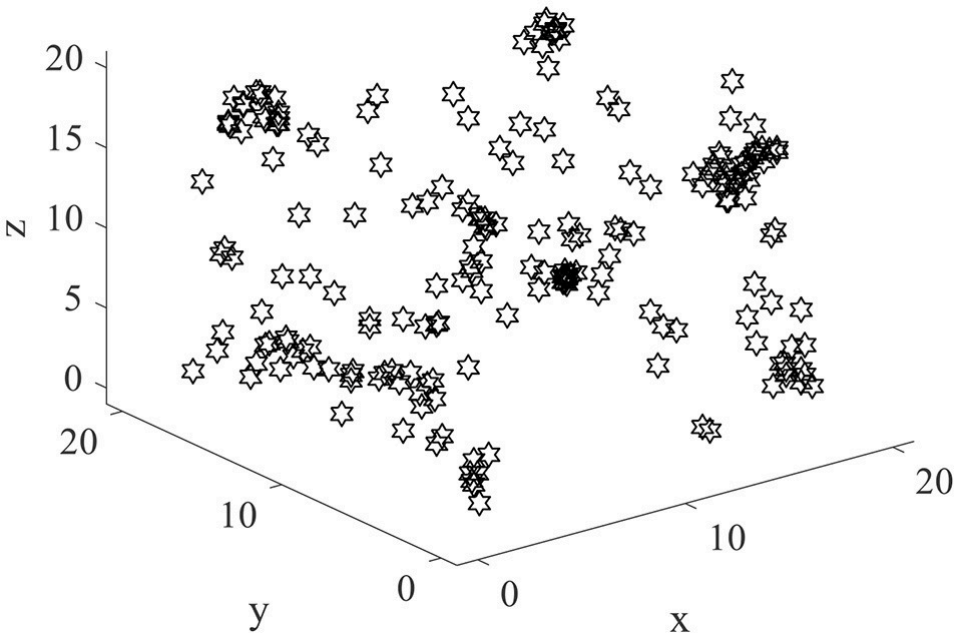
The distribution of place field centers in three dimensional space.

Normally the place field centers are near the locations with maximum powers. Two hundred place field centers are summarized in Figure 7. As can be seen from the figure, the 200 field centers are scattered throughout the space. The phenomenon mentioned by Kulvicius et al. (2008) that place field centers may concentrate near the spatial center is not occurred. The density of place field centers is quite uniform. Plenty of place field centers are near the space borders.

**Figure 8:**
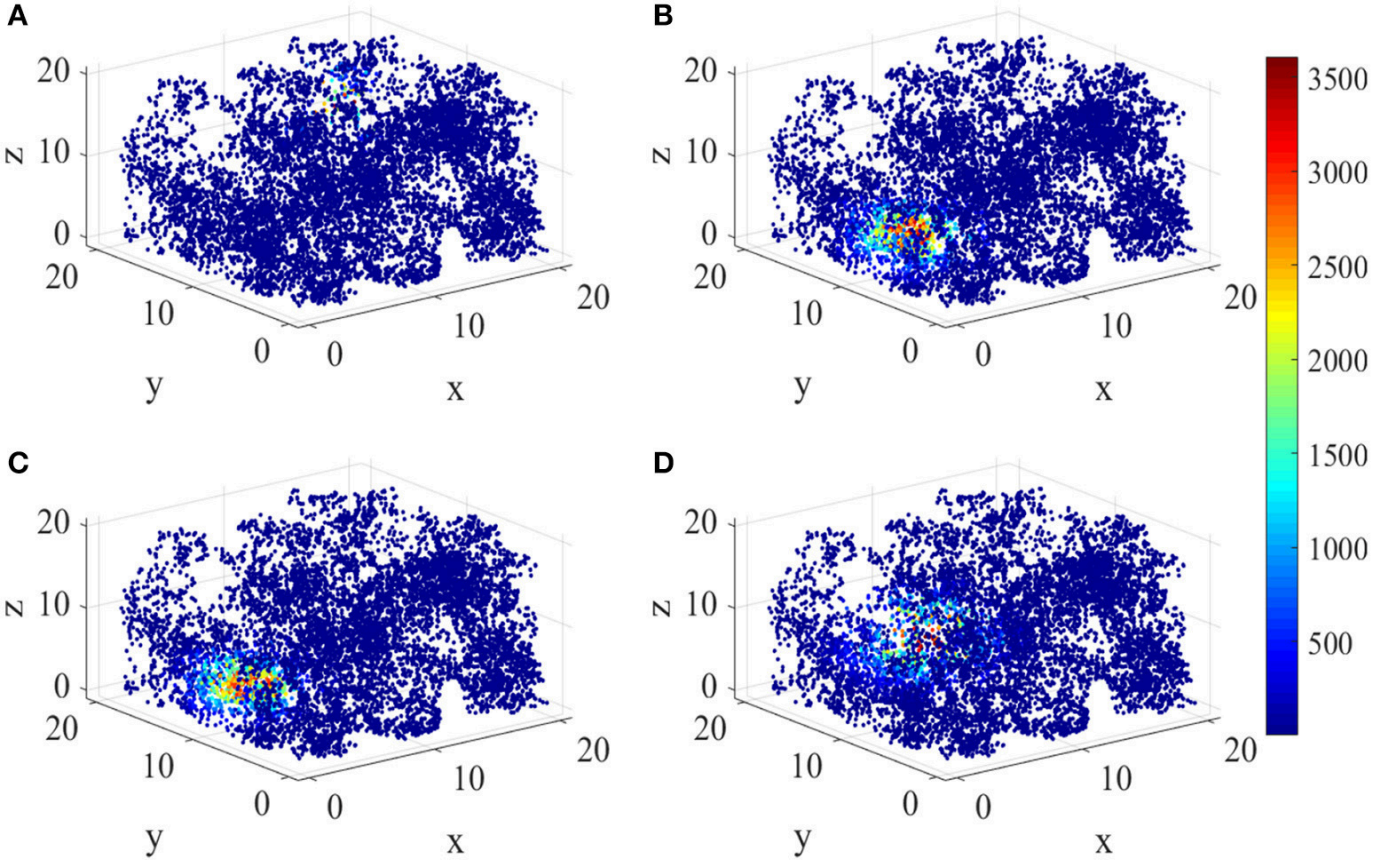
Spatial selectivity of place cells. **(A–D)** are place fields of four randomly selected cells numbered 40, 163, 50, 81 respectively. They have various responding locations and different sizes.

Figure 8 shows the energy place fields of four randomly selected place cells. From this figure, the individual differences and the unique spatial selectivity are clearly revealed. Place cell a has a smaller place field near wall B, R, and U. Place field of cell b is larger, which is one of the neurons with higher energy consumptions.

### Locating error and energy consumption of the place cell network in three dimensional space

**Figure 9:**
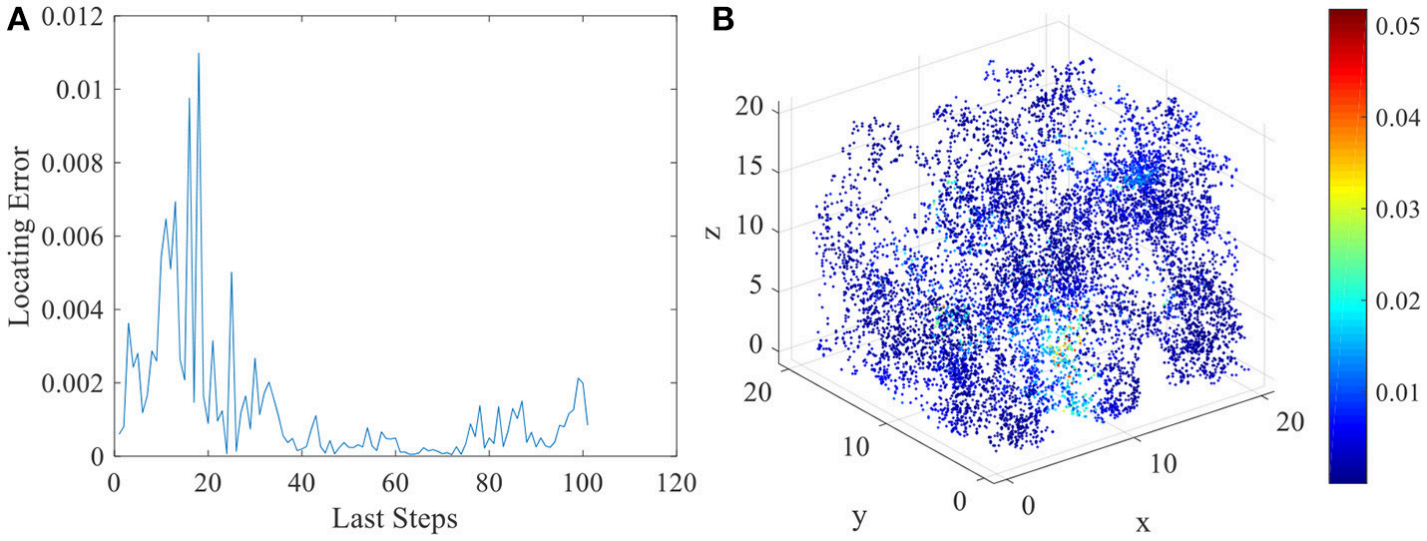
Locating errors in three dimensional space of place cells. **(A)** shows the relative locating errors of the last 100 steps of the exploration. **(B)** shows all the relative locating errors of the 10000 exploration steps.

The various spatial selectivity and the corresponding place fields indicate that the model simulated the basic features of place cells in three dimensional space. As soon as the place fields are formed and field centers are defined, the locating function of the network can be performed. As described in section Energy Model for Place Cells and Learning Rule, the location of the animal will be determined by the weighted average of place field centers belong to the response set. We compared the locating results and the actual spatial positions of the animal to analyze the locating error of this network model. In Figure 9A, we chose the last 100 steps during the random exploration and calculated the locating errors for these 100 locations. As the figure shows, the error between locating and actual position fluctuates under an acceptable low level. Errors of all 10,000 steps during the exploration are shown in Figure 9B.

Two sources account for the locating error. One is the systematic error of the network model. This is a model using a finite number of place fields to determine infinite even uncountable infinite number of locations in three dimensional space. This will cause inevitable error. And the degrees of freedom for spatial location is three, but after receiving three sensory inputs, place cell integrates this three dimensional information into one dimensional variable, which is firing power. Restoring the three dimensional information from one will clearly cause error. This is systematic error for the model. The other source of error is the inaccuracy of sensory. In order to simulate the inaccurate estimation of distance to landmark of the animal, we add the error term to the sensory model. This will also lead to the locating error. Excluding this term could reduce the locating error to a lower level. However, the final locating errors are limited under a certain boundary. So this network model can successfully perform the locating function in three dimensional space.

**Figure 10:**
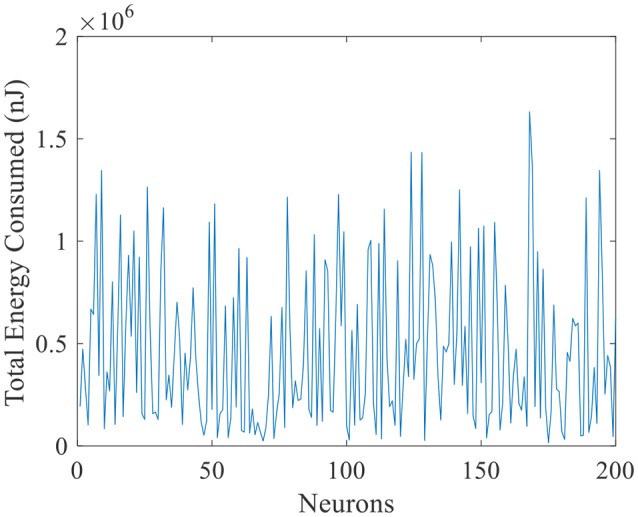
Total energy consumption during exploration of place cells.

The total energy consumed by these 200 place cells during the exploration process is illustrated in Figure 10. The horizontal axis is number of place cells, and vertical axis is the total energy consumption. The maximum energy consumed by a single cell is close to 1.7 × 10^6^ nJ. And the minimum is close to zero. Many cells remain a low energy cost while preforming the locating function, this implies that during the spatial representation process, the neural system complies with the energy efficiency principle. It means to code the neural information with minimum energy consumption (Wang R. et al., 2015).

### The impact on locating error, energy consumption, and distribution of place field size

**Figure 11:**
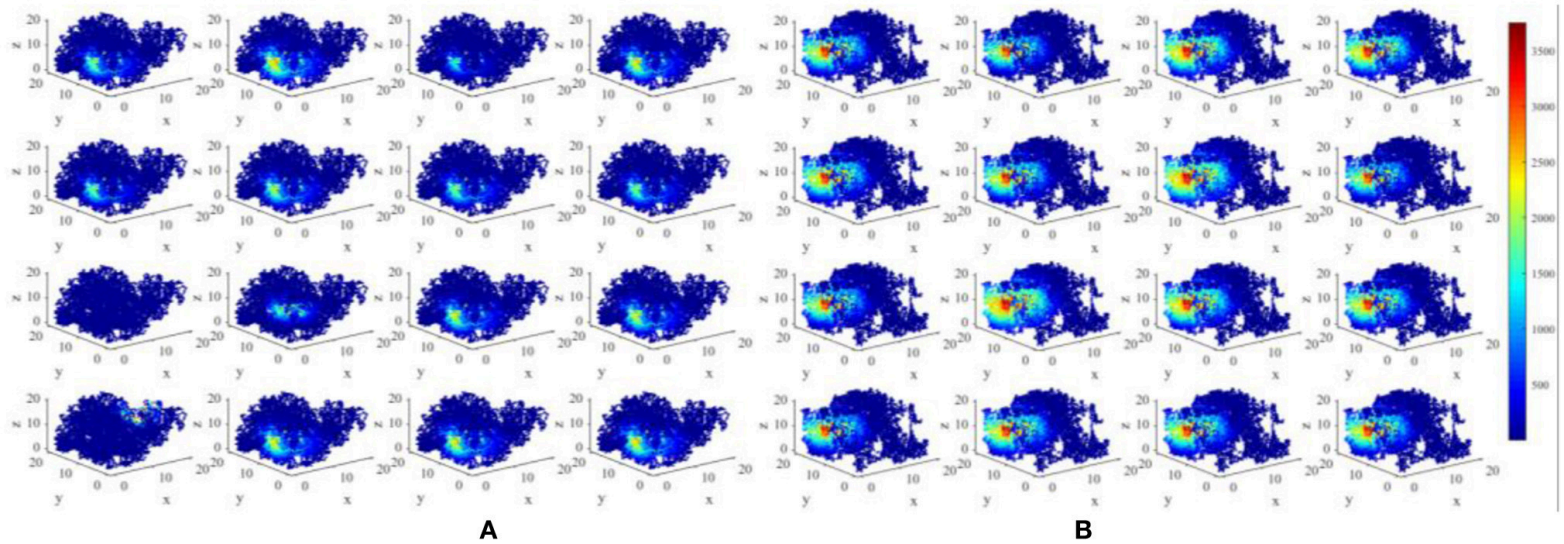
Larger place fields and higher energy consumptions. **(A)** Spatial responses and energy consumption of slightly larger place fields of 16 randomly selected cells. **(B)** Spatial responses and energy consumption of much larger place fields of 16 randomly selected cells. The enlargement the place fields can expand the spatial range of high power activity of place cells.

We have constructed an energy model of place cells to perform the locating function in three dimensional space. In this model, an important parameter is σ_j_, as mentioned in section Model and Method, which is a random variable complying with Gaussian distribution, which influences the size of place fields (Kulvicius et al., 2008). The expectation of this distribution affects the mean size of place fields, and the standard deviation affects the variability of different place fields. Figure 11 depicts two groups of place fields with greater size. As showed in the figure, the enlargement the place fields can expand the spatial range of high power activity of place cells (Figure 11B). Notably, the field size and selectivity will still evolve dynamically as the exploration and learning proceed. While the place fields are too large, place fields belong to different cells will have more overlapped part. During coding a position in space, several place cells will be activated at the same time and are more likely to response with higher powers. This is not economical or reasonable from the energy perspective. So the spatial representation system of the brain is possible the balancing result between spatial coverage (accuracy) and energy efficiency.

**Figure 12:**
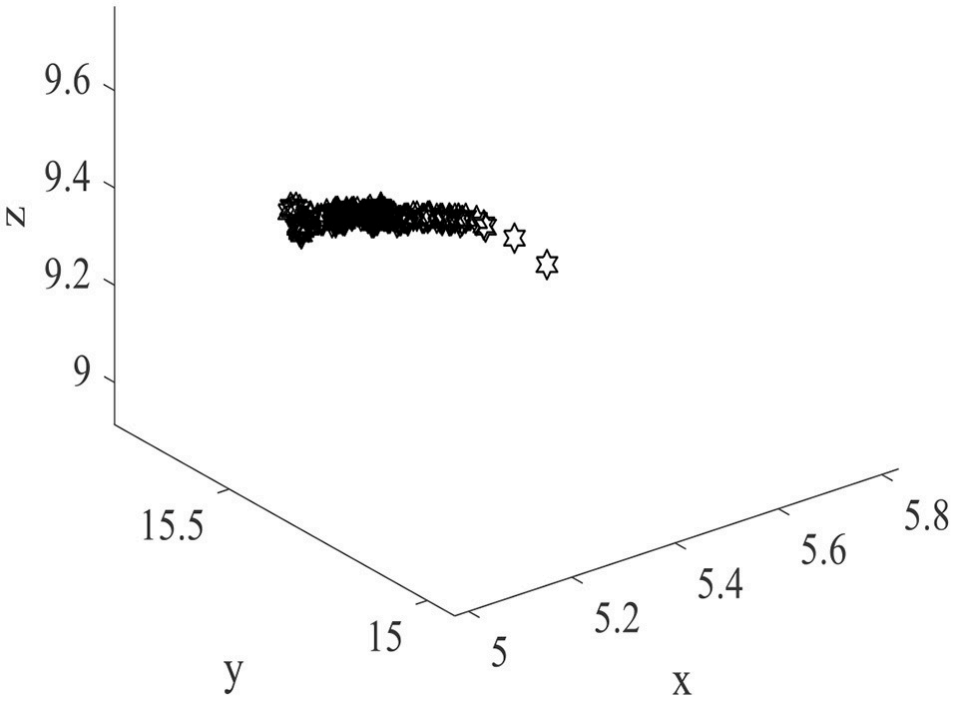
Linear correlation of place field centers.

Meanwhile, higher energy consumption and larger place fields imply that during the spatial learning process, more place cells obtain highly overlapping place fields, place cells with similar initial weights may become more alike, resulting in the correlations of place fields become higher. This phenomenon is shown in Figure 12. Two hundred field centers show a strong linear correlation, and concentrate in the center of space. This suggests that in the case of high power consumption, place cells have a high redundancy coding the spatial information and consume too much unnecessary energy. This result once again confirms the economic principle of the energy usage in the information coding of the brain.

**Figure 13:**
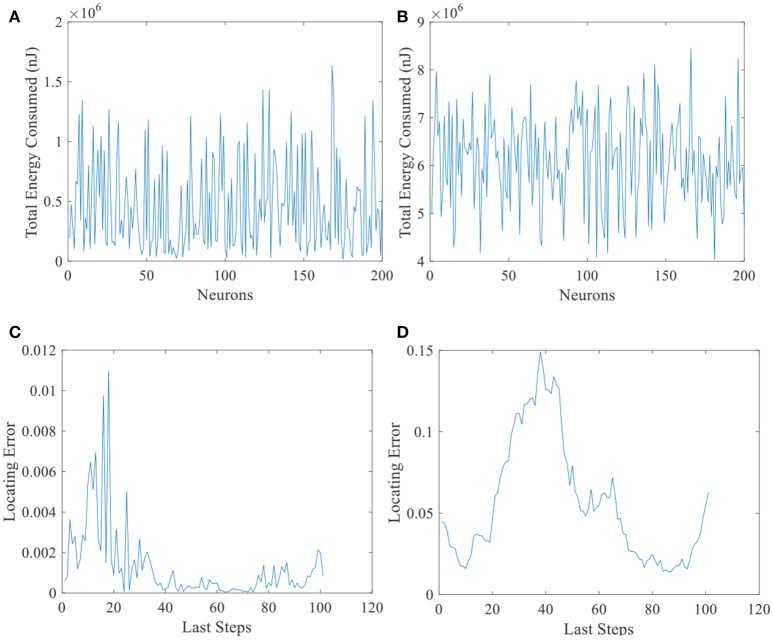
Energy consumed and locating errors. Total energy consumption **(A)** and the locating errors of the last 100 steps **(C)** of 200 place cells with smaller place fields. **(B)** and **(D)** are similar with **A** and **C** except that the size of place field is larger.

Another evidence can be seen in Figure 13. Total energy consumed by 200 place cells with larger place fields (Figure 13B) and the corresponding locating errors (Figure 13D) are shown in this figure. Unlike Figure 13A, all the cells consumed more than 4 × 10^6^ nJ energy, while the locating errors are larger than the small field situation (Figure 13C). This suggests that the energy consumption in the neural system is not *the more, the better*.

**Figure 14:**
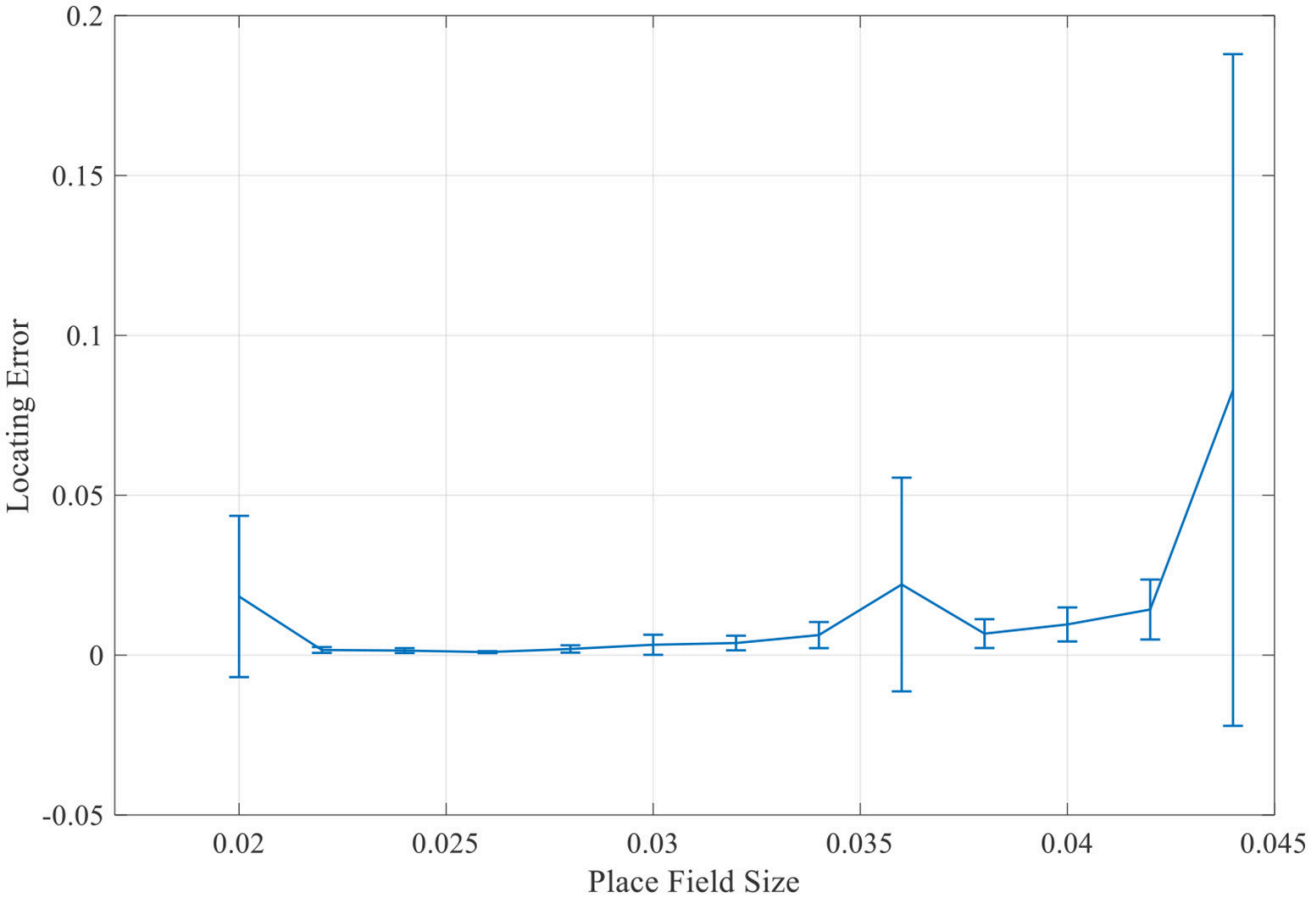
Mean locating errors with respect to place field sizes.

More simulations with a larger range of place field sizes suggest that the final locating error was not simply monotonously increasing as the place field enlarging. There always exists a minimum localization error when the place field is of the medium size (Figure 14). So the place field with a reasonable optimal size will most accurately preform the localization function.

**Figure 15:**
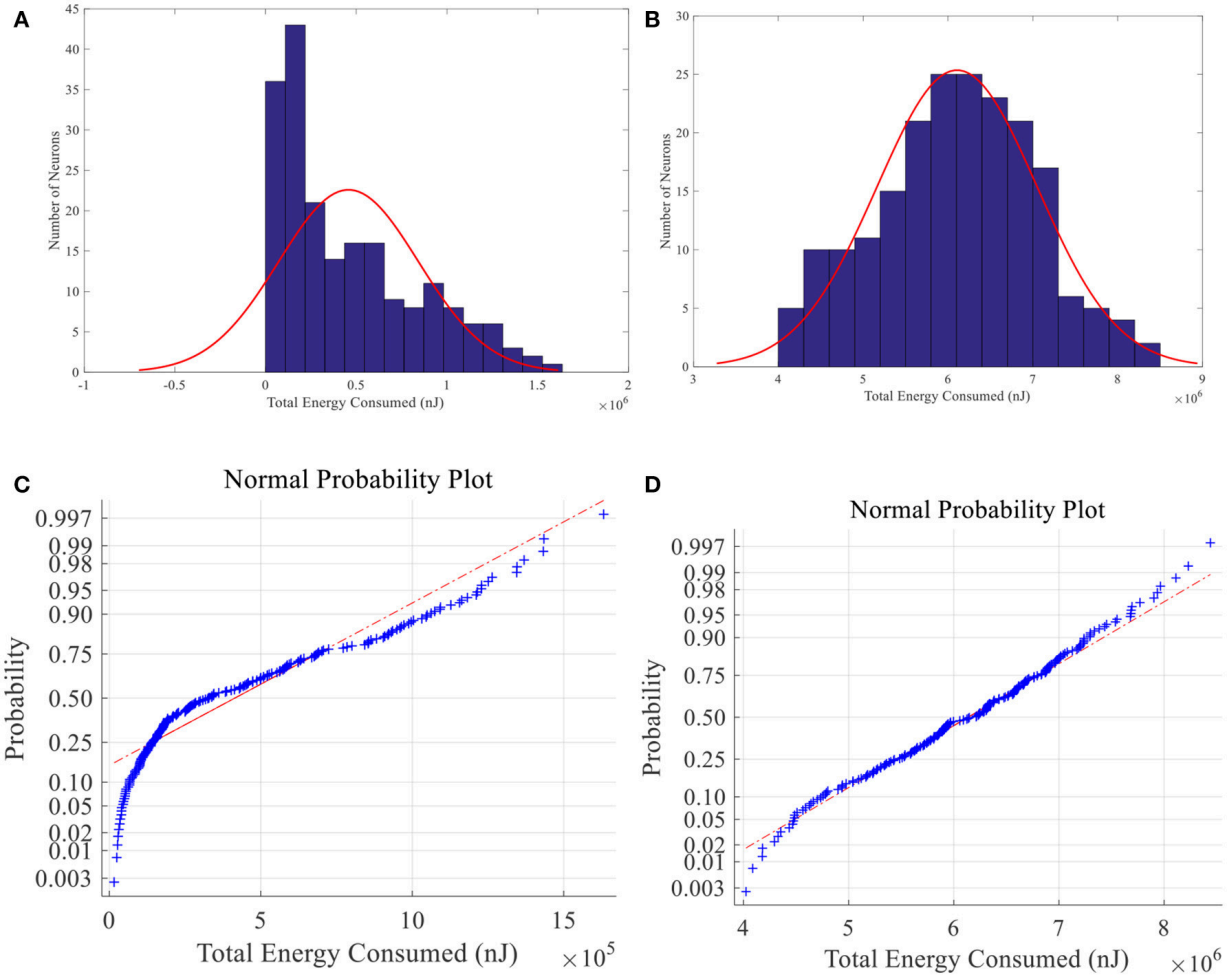
Total energy consumption distribution of place fields. Histogram **(A)** and normal probability plot **(C)** of total energy consumption of small size place fields, which are not normally distributed. **(B)** and **(D)** have the same meaning with **A** and **C** except that the place fields are larger and the energy consumption is normally distributed.

When place field is small, the total energy consumption is not normally distributed among 200 place cells whereas the normal distribution hypothesis is failed to be rejected in large-place-field situation (α = 0.01) (See Figure 15). Energy of larger place field cells is closer to normal distribution. Since information can be seen as the degree of unexpectedness, it will not be totally random. So the difference between neural energy distribution and the normal distribution may be crucial to understand neural information coding. And cells with moderate smaller place fields consumed less energy possibly contain more spatial information (Brown and Backer, 2006).

### Experiment support to the model results

**Figure 16:**
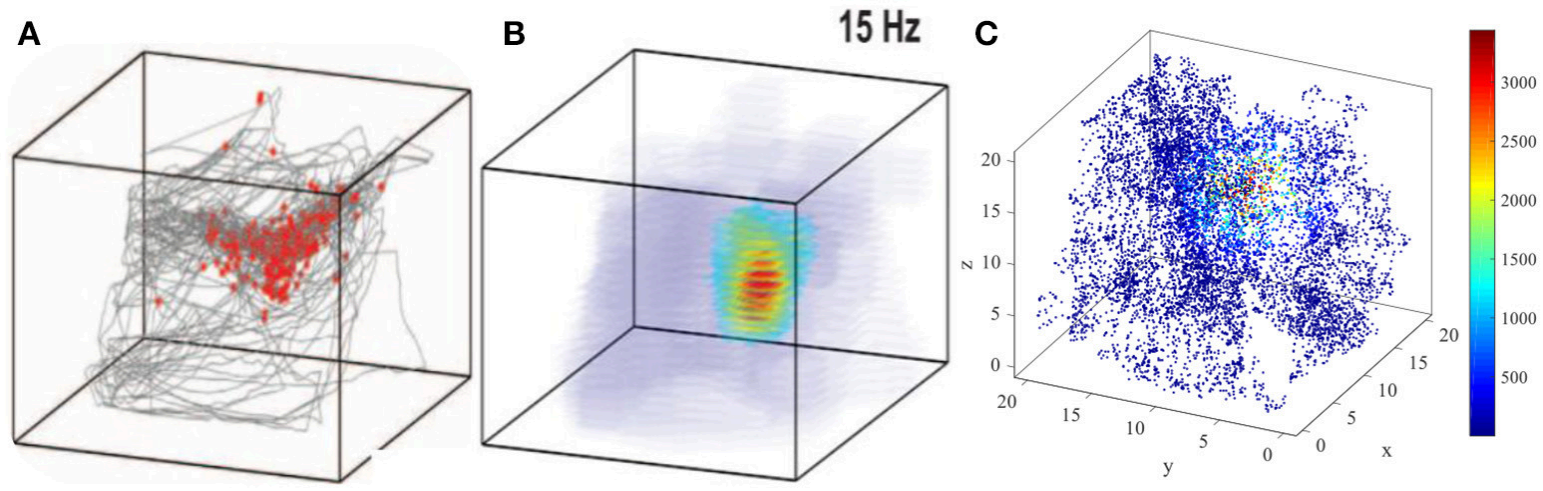
Model result compared with experimental recording (Yartsev and Ulanovsky, 2013). **(A)** and **(B)** show the experiment results from the work of Yartsev and Ulanovsky. **(A)** is the spikes (red dots) overlaid on bat's flying trajectory (gray lines). **(B)** is the three dimensional color coded rate map, with peak firing rate of 15 Hz. **(C)** is the model simulation result of the typical activity pattern of a place cell.

The three dimensional spatial tuning of this network model is comparable with the valuable experiment recordings. Figure 16 illustrate the comparison between model result and experiment data recorded from bat (Yartsev and Ulanovsky, 2013). Figure 16A shows the spikes (red dots) overlaid on bat's position (gray lines), and Figure 16B is the three dimensional color-coded rate map, with peak firing rate of 15 Hz. Figure 16C is the model simulation of the typical activity pattern of a place cell. By comparing these figures we can find out that the simulation result is similar with the experimental result morphologically. In this point of view, the behavior of this model is matched with the experimental data.

## Discussion

Energy efficiency is one of the most remarkable features of the neural systems. In mammalian brains, 1,000 trillion operations per second are carried out while only several watts of energy is consumed (Kandel et al., 2000). This feature should be addressed while modeling the function of brain. As a scalar and a more fundamental physical variable than others such as spike number, firing rate, or oscillation phase, neural energy has been proved to be an effective tool for neural modeling and computational analyzing due to its global and multi-level superimposing properties, which will greatly reduce the cost of analytical research (Wang et al., 2017b; Zhu et al., 2018). Using energy rather than firing rate model for place cell will emphasize cognitive function as well as neural cost and the tradeoff between these two aspects can be revealed. It will help us verify the principle of minimizing the energy consumption while maximizing the signal transmission efficiency in the brain (Laughlin and Sejnowski, 2003). Besides, energy provides a mutual code for neural activities on every level from ion channels to global brain. This energy model will be conveniently embedded into a more macroscopic model unified by energy.

Energy consumption is positively related to the place field size. If the place field is too small, the locating system will fail to cover the local space and provide spatial information insufficiently. On the contrary, large place field will convey adequate even redundant spatial information at the cost of much more energy consumption. So the locating system has to balance the spatial coverage and energy consumption, which leads to a moderately medium place field size as this model shows. And the balanced field size and energy consumption jointly regulate a more accurate locating function. A coupling energy model of grid cell and place cell could be constructed in future study, which will help us understand the energy efficiency principle in medial entorhinal cortex-hippocampus circuit and further the whole spatial cognition system of the brain.

This is a preliminary model for three dimensional spatial representation system and certain factors are simplified. For example, it is known that animal rely on visual, auditory, olfactory, or somatosensory stimuli for orientation. While in this model, the sensory input to place cell network is in an abstract form without addressing the type of the cues. And the neural energy consumed by synaptic transmission is neglected in this model for simplification. These shortages will be interesting topics for future modeling study. However, this model, which emphasizes the three dimensional locating function and takes energy efficiency into consideration as well, may be the initial step to complete a comprehensive energy model for the brain's spatial representation system in realistic three dimensional world.

## Conclusion

Aiming at improving the defects of the studies of place cells and energy coding, we constructed a place cell network model representing three dimensional space on an energy level. Then we defined the place field, place field center by energy. The spatial representation and locating functions of this model have been analyzed and the energy consumption properties related to place fields and locating accuracy have been studied. The computational results showed that the model successfully simulated the basic features of place cells. The spatial selectivity and sizes of place fields vary among individual place cells, and the locating error can be limited under an acceptable low level by choosing the reasonable parameters. Then we demonstrated the relationship between energy consumption, place field size, and locating error. Furthermore, we found that the minimum value of locating error will be obtained when the place field is of moderate small size. This may suggest that the place cell network balance the spatial coverage and the energy consumption to achieve an accurate locating function, which implied the energy efficiency feature of the neural systems. The simulation results matched with experimental data (Yartsev and Ulanovsky, 2013). In conclusion, this is an effective model to represent three dimensional space by energy method. It is a generalization model for higher dimensional space on a more fundamental energy level. The research verifies the energy efficiency principle of the brain during the neural coding for three dimensional spatial information. It is the preliminary step to complete the model of three dimensional spatial representing system of the brain, and will help us further understand how the brain's locating, navigating and path planning function are performed in the realistic three dimensional space.

Besides the locating function, path-planning and navigation are also the crucial functions of the brain's spatial representation system. Other cells such as grid cell, border cell, and head-direction cell should be introduced in future studies. These models of different types of cells can also be generalized into three dimensional space by this similar energy method. Then the system error of locating may be reduced significantly and the model will acquire the path integrating and navigation function in three dimensional space. Moreover, whether the degree of freedom of the sensory input is higher in three dimensional space than on a plane remains to be testified by physiology experiments. If it can be verified that there is a certain group of cells in the brain responding solely to altitude information, the dimension of integrated signals can be extended and the locating accuracy can be improved in the model. These future works will help us understand and explain the three dimensional spatial representation system of the brain, and will further reveal how the energy efficiency principle would guide the brain to execute the locating, path planning and navigating functions. It will be a new view to study the mystery of the brain.

## Author contributions

YW: co-designed the research, constructed the model, wrote software code, wrote paper; XX: co-designed the research, analyzed results, contributed to writing of software code and paper; RW: participated in research design, model construction and results analysis, edited paper.

### Conflict of interest statement

The authors declare that the research was conducted in the absence of any commercial or financial relationships that could be construed as a potential conflict of interest.
